# Ibuprofen Ingestion Does Not Affect Markers of Post-exercise Muscle Inflammation

**DOI:** 10.3389/fphys.2016.00086

**Published:** 2016-03-29

**Authors:** Luke Vella, James F. Markworth, Gøran Paulsen, Truls Raastad, Jonathan M. Peake, Rod J. Snow, David Cameron-Smith, Aaron P. Russell

**Affiliations:** ^1^Centre for Physical Activity and Nutrition Research, School of Exercise and Nutrition Science, Deakin UniversityBurwood, VIC, Australia; ^2^Liggins Institute, University of AucklandAuckland, New Zealand; ^3^Department of Physical Performance, Norwegian School of Sport ScienceOslo, Norway; ^4^School of Biomedical Sciences and Institute of Health and Biomedical Innovation, Queensland University of TechnologyBrisbane, QLD, Australia

**Keywords:** exercise recovery, NSAID treatment, inflammation, resistance exercise, leucocyte

## Abstract

**Purpose:** We investigated if oral ingestion of ibuprofen influenced leucocyte recruitment and infiltration following an acute bout of traditional resistance exercise

**Methods:** Sixteen male subjects were divided into two groups that received the maximum over-the-counter dose of ibuprofen (1200mg d^−1^) or a similarly administered placebo following lower body resistance exercise. Muscle biopsies were taken from m.vastus lateralis and blood serum samples were obtained before and immediately after exercise, and at 3 and 24 h after exercise. Muscle cross-sections were stained with antibodies against neutrophils (CD66b and MPO) and macrophages (CD68). Muscle damage was assessed via creatine kinase and myoglobin in blood serum samples, and muscle soreness was rated on a ten-point pain scale.

**Results:** The resistance exercise protocol stimulated a significant increase in the number of CD66b^+^ and MPO^+^ cells when measured 3 h post exercise. Serum creatine kinase, myoglobin and subjective muscle soreness all increased post-exercise. Muscle leucocyte infiltration, creatine kinase, myoglobin and subjective muscle soreness were unaffected by ibuprofen treatment when compared to placebo. There was also no association between increases in inflammatory leucocytes and any other marker of cellular muscle damage.

**Conclusion:** Ibuprofen administration had no effect on the accumulation of neutrophils, markers of muscle damage or muscle soreness during the first 24 h of post-exercise muscle recovery.

## Introduction

Unaccustomed resistance exercise often results in tissue damage and inflammation, leading to delayed onset muscle soreness (DOMS) and a consequent reduction in force production (Faulkner et al., 1993; Tidball, 1995). Animal models and *in vitro* studies have identified that local and systemic inflammation exerts a regulatory influence during the different phases of muscle recovery, including myofibrillar disruption, cellular necrosis, satellite cell activation, maturation and subsequent regeneration, and adaptation (Fridén et al., 1981; Armstrong et al., 1991). Thus, post-exercise inflammation is intimately necessary and a key feature of the normal process of tissue regeneration and adaptation following acute muscle damage. However, excessive inflammation is considered a potential cause of prolonged post-exercise muscle soreness and may have a negative effect on muscle recovery (Armstrong et al., 1991; Smith, 1991); consequently strategies to reduce or counteract inflammation are commonly implemented to aid in improving muscle recovery after exercise (Urso, 2013).

Non-steroidal anti-inflammatory drugs (NSAIDs) are commonly used as a treatment strategy in exercise and sports medicine to assist with recovery from exercise-induced inflammation, particularly following soft-tissue injury. NSAIDs inhibit the cyclooxygenase (COX-1 and 2) enzymes and consequently the formation of prostanoids (prostaglandins, prostacyclins, and thromboxanes) that play a diverse role in acute inflammation (Markworth et al., 2013). Prostanoids stimulate an acute inflammatory process by controlling local blood flow, vascular permeability, leucocyte infiltration, and triggering sensations of pain (Markworth et al., 2013; Urso, 2013). In animal models of acute muscle damage, treatment with NSAIDs blunts the infiltration of leucocytes into muscle tissue (Lapointe et al., 2003; Bondesen et al., 2004) and causes a reduction in creatine kinase (CK) (Mishra et al., 1995). Consequently, NSAIDs can also inhibit myofiber regeneration, satellite cell proliferation and differentiation, and overload-induced muscle hypertrophy (Mishra et al., 1995; Bondesen et al., 2004, 2006). These findings provide preliminary evidence that NSAIDs compromise the physiological link between processes of acute muscle damage, inflammation and cellular regeneration.

Research into the effects of NSAIDs on exercise-induced muscle damage and inflammation has produced equivocal findings. In exercise models, NSAID administration has been shown to attenuate post-exercise DOMS in some (Baldwin et al., 2001; Tokmakidis et al., 2003; Paulsen et al., 2010), but not all studies (Trappe et al., 2002; Krentz et al., 2008; Mikkelsen et al., 2009; Hyldahl et al., 2010). While a precise cellular mechanism for an analgesic effect of NSAIDs remains unclear, it has been largely ascribed to their effect on prostaglandin synthesis and the capacity of NSAIDs to interfere with aspects of inflammatory cell function (Hersh et al., 2000; Peterson et al., 2003). Previous research has demonstrated that oral consumption of both ibuprofen, a non-selective NSAID and acetaminophen, an analgesic also known as paracetamol, had no effect on macrophage infiltration at 24 h following an eccentric exercise protocol (Peterson et al., 2003). Similarly treatment with naproxen, another non-selective NSAID, had no effect on the infiltration of leucocyte common antigen positive cells following a unilateral, isotonic resistance exercise protocol (Bourgeois et al., 1999). Interestingly, Paulsen et al. (2010) suggested a blunting effect of the COX-2 specific celecoxib following maximum eccentric muscle contractions (Paulsen et al., 2010). This research identified a tendency for higher monocyte/macrophage numbers in subjects within the placebo group, and those subjects who were identified as “high-responders” to the exercise protocol based on the number of inflammatory leucocytes (Paulsen et al., 2010). Although this is not a particularly robust finding, it has led to the hypothesis that NSAIDs may influence leucocyte infiltration in skeletal muscle when a sufficiently strong and early inflammatory reaction is present (Paulsen et al., 2010). This hypothesis would suggest that the intensity of the exercise stimulus and the consequent muscle-damage response would be largely influential in determining the effect of a pharmacologically based anti-inflammatory intervention.

Conflicting findings also exist with regard to the effect of NSAIDs on the regenerative capacity of skeletal muscle following exercise-induced muscle damage. The non-selective COX inhibitor ibuprofen blunted skeletal muscle protein synthesis (Trappe et al., 2002) while local intramuscular infusion of indomethacin (Mikkelsen et al., 2011) and the oral administration of the COX-2 selective NSAID celecoxib (Burd et al., 2010; Paulsen et al., 2010) had no such effect. Similarly, treatment with indomethacin inhibited post-exercise satellite cell proliferation (Mikkelsen et al., 2009). Recent work from our group demonstrated that treatment with ibuprofen inhibited early translational signaling responses involved in post-exercise muscle hypertrophy (Markworth et al., 2014). A clear mechanistic pathway for NSAIDs to influence the physiological link between post-exercise inflammation and skeletal muscle regeneration remains elusive.

These discrepancies in the research to date are likely due to differences in the exercise protocol (concentric vs. eccentric muscle contractions), the training status of subjects, the timing of muscle biopsies, and the type of NSAID, the dosage administered, and method of administration. Further research is required to determine whether NSAID administration affects the infiltration of leucocyte populations following exercise-induced muscle damage and how this influences post-exercise adaptive pathways. The aim of the present study was to investigate if oral administration of the NSAID ibuprofen influenced skeletal muscle leucocyte infiltration during the first 24 h after muscle-damaging resistance exercises. We also aimed to explore how any changes in leucocyte infiltration were related to markers of muscle damage, including circulating muscle proteins CK and myoglobin, and subjective markers of muscle soreness. We hypothesized that the ingestion of ibuprofen following acute resistance exercise would not influence the infiltration of leucocytes, however it would attenuate sensations of DOMS.

## Materials and methods

### Participants

As described previously, 16 healthy male subjects were recruited to participate in the study (Table 1; Markworth et al., 2013). It is important to note that the purpose of the original study by Markworth et al. (2013) was to profile the human eicosanoid response to acute exercise in blood plasma samples, as opposed to the aim of the present study which was to determine the effect of oral ingestion of ibuprofen on leucocyte recruitment and infiltration following an acute bout of traditional resistance exercise. While the subjects and experimental design are the same in both studies the aims and dependent variables reported are different. Exclusion criteria included participation in a lower body resistance exercise program within the last 6 months to ensure a muscle damage response from the exercise stimulus, and/or previous chronic treatments with anti-inflammatory medication. Participants also completed a medical screening to identify any potential risk factors for them to perform strenuous physical activity.

**Table 1:** **Subject characteristics and strength testing data**.

	**Characteristics**				**Strength (1 RM)**		
	**Age (y)**	**Height (m)**	**Body mass (kg)**	**BMI**	**Squat (kg)**	**Leg press (kg)**	**Leg extension (kg)**
PLA	23.9 ± 1.3	1.89 ± 0.1	86.9 ± 4.5	24.5 ± 1.2	94.9 ± 5.4	237 ± 17	236 ± 18
IBU	23 ± 0.5	1.89 ± 0.1	89.1 ± 4.4	24.8 ± 0.8	91.9 ± 6.0	240 ± 15	196 ± 22

*Values are mean values ± SEM. No significant differences were observed between the two groups. PLA, placebo; IBU, Ibuprofen; BMI, body mass index*.

### Ethics approval

All procedures involved in this study were approved by the Deakin University Human Research Ethics Committee (DUHREC 2010-019) and muscle sampling procedures were performed in accordance with the Helsinki declaration. Each participant was provided with written and oral details of the nature and requirements of the study and provided written consent to participate.

### Familiarization

Each participant completed a familiarization and strength testing session at least 7 days prior to completing the exercise protocol. Details of the familiarization session have been described previously (Markworth et al., 2013). Briefly, subjects performed repetition maximum testing for the Smith machine-assisted squat, the leg press and the leg extension to determine their experimental exercise load [80% of a 1 repetition maximum (1 RM)]. The maximum weight the subject could lift for 5–8 reps was determined, and these data were entered into the Brzycki equation to predict 1 RM (Whisenant et al., 2003). Subjects were asked to abstain from any further activity until the completion of the trial.

### Experimental procedures

The participants reported to the laboratory on the morning of the trial in an overnight fasted state. They were asked to abstain from caffeine, tobacco and alcohol for the 24 h preceding the trial. Participants rested in a supine position for 30 min, following which the first muscle biopsy sample was taken. Each participant then completed a 10 min warm-up protocol comprised of 5 min of low intensity cycling on a stationary bike, and one low-intensity set of each exercise at a weight of each subject's own choice within the range of 30–50% 1 RM. The resistance exercise session consisted of three sets of 8–10 repetitions performed on a Smith machine assisted squat, a 45° leg press and a leg extension at 80% of a predicted 1 RM. The exercises were performed as a circuit with 1 min rest permitted between exercises and 3 min rest between sets. This protocol has been used previously and has been a sufficient stimulus to activate inflammatory signaling pathways (Vella et al., 2012) and was implemented to replicate a commonly used exercise routine. After exercise, the subjects rested while subsequent muscle biopsy samples were collected. After the 3 h biopsy, participants were provided a standardized meal and were allowed to go home. The following morning, participants reported to the laboratory in an over-night fasted state for a 24 h muscle biopsy and blood sample.

### Standardized meals

Standardized meals were provided to participants on the night before the trial, (carbohydrate 57%, fat 22%, protein 21%), immediately following the 3 h muscle biopsy (carbohydrate 71%, fat 13%, protein 18%), and in the evening (carbohydrate 64%, fat 27%, protein 18%) on the day of the exercise trial. Participants were permitted to drink water *ad libitum* and were asked to report if they could not finish their allocated meals. This nutrition plan was included to ensure that each participant received the same relative percentage of macro- and micro-nutrients.

### NSAID administration

Prior to exercise, participants were randomly assigned in a double-blind method, to consume either the maximum recommended dose of ibuprofen (IBU, *n* = 8) or a placebo control (gelatin capsules identical in appearance containing powdered sugar in place of ibuprofen) (PLA, *n* = 8). The IBU group consumed three doses of 400 mg of ibuprofen throughout the trial day. The first dose was administered immediately prior to the first muscle biopsy sample. Participants were instructed to consume the following two doses at 6 and 12 h following the exercise protocol. Follow-up phone calls from the research team ensured compliance. To ensure this dosing structure was appropriate to maintain biologically active levels of ibuprofen, ibuprofen concentration was measured in blood serum samples and these data are reported elsewhere (Markworth et al., 2013).

### Sample collection

Venous blood samples were drawn prior to exercise, within 5 min post exercise (here-after referred to as 0 h post-exercise), and at 1, 2, 3, and 24 h post-exercise. Blood samples were drawn through an indwelling catheter into VACUETTE serum tubes (Greiner, Bio-One, Stonehouse UK). Whole blood was allowed to clot at room temperature. Samples were then centrifuged at 1000 × g for 10 min. The serum layer was collected and stored at −80°C for subsequent analysis.

Muscle biopsy samples were obtained from the vastus lateralis muscle under local anesthesia (Xylocaine 1%) using a percutaneous needle biopsy technique modified to include suction (Buford et al., 2009). To minimize interference from the biopsy procedure, samples prior to exercise and at 24 h post exercise were taken from the same leg, while samples taken at 0 and 3 h post-exercise were taken from the contralateral leg. Samples obtained within the same leg were taken at least 5 cm from the previous incision site. We have previously reported that this technique is effective for minimizing the risk of any inflammation arising from the biopsy procedure confounding exercise-induced inflammation (Vella et al., 2012). Tissue-Tek immersed tissue was rapidly frozen in isopentane cooled liquid nitrogen before storage at −80°C.

### Immunohistochemistry

Cross sections (8 μm) of muscle tissue were cut using a microtome at −20°C (CM3050, Leica, Germany) and mounted on microscope slides (Superfrost Plus, Thermo Scientific, MA, USA), air-dried and stored at −80°C. Each subject's muscle sections from all time points were mounted on the same microscope slide. Serial muscle cross-sections were stained with three different leucocyte antibodies, MPO (#0398, DakoCytomation, Glostrup, Denmark; dilution 1:2000), CD66b (#CLB-B13.9, Sanquin Reagents, Amsterdam, The Netherlands; dilution 1:500), and CD68 (#EBM-11, DakoCytomation, Glostrup, Denmark; dilution 1:300). Furthermore, the sections were also stained with dystrophin antibody (ab15277, Abcam, Cambridge, UK) for visualizing borders of muscle fibers.

Sections were fixed in 4% paraformaldehyde solution in a staining jar for 5 min at room temperature and rinsed twice for 10 min in phosphate buffered saline (PBS) containing 0.5% Tween 20 (Sigma-Aldrich). The microscope slides were moved into a humidified chamber and non-specific binding sites were blocked with 1% bovine serum albumin (BSA) on the section for 45 min at room temperature. Sections were incubated overnight with leucocyte and dystrophin antibodies diluted in 1% BSA at 4°C. After overnight incubation, the slides were washed three times for 10 min in PBS-Tween in a staining jar. Slides were moved back to the humidified chamber and sections were incubated for 45 min with secondary antibodies diluted 1:200 in 1% BSA at room temperature. Alexa Fluor® 594 F(ab′)2 fragment of goat anti-rabbit and Alexa Fluor®488 anti-mouse IgG (Invitrogen, Eugene, Oregon, USA) were used as a secondary antibodies. The fluorochrome-stained sections were washed three times for 10 min in PBS-Tween. After the last washing in PBS-Tween, the sections were mounted on ProLong® Gold Antifade reagent with DAPI (Invitrogen, Eugene, Orgeon, USA).

Muscle sections were visualized using a high-resolution camera (DP72, Olympus, Japan) mounted on a microscope (BX61, Olympus, Japan) with a fluorescence light source (X-Cite 120PCQ, EXFO, Canada). The number of MPO, CD66b, and CD68 positive cells (as well as the total number of muscle fibers from the area included) was counted. Data was calculated as percentage of MPO, CD66b, or CD68 positively stained cells per 100 skeletal muscle fibers. Areas of sections that contained freeze damage or were folded due to the cutting procedure were not included in the analysis.

### Biochemical assays

Serum creatine kinase activity in pre- and post-exercise blood samples was analyzed using an enzymatic assay (CK-NAC kit, CDT14010, Thermo-Fisher Scientific Clinical Diagnostics, Sydney, Australia) and an automated clinical analyser (Cobas Mira, Roche Diagnostics, Germany). Serum myoglobin concentration was also measured in these samples using an immunoassay (Roche Diagnostics, Germany) and an automated clinical analyzer (Cobas E411, Roche Diagnostics, Germany). The intra-assay coefficient of variation was 10.4% for creatine kinase and 1.7% for myoglobin.

### Muscle soreness assessment

Upon arrival at the laboratory and prior to the first muscle biopsy procedure, as well as 24 h following exercise prior to the fourth and final muscle biopsy, subjects were asked to rate their subjective muscle soreness on a 0–10 visual analog scale. The subjects were instructed to contract, stretch, and palpate the quadriceps muscle to assess general muscle soreness. In both instances 0 was considered to represent no pain and correspondingly a rating of 10 was considered to represent intense pain.

### Statistics

Data are expressed as means ± SEM. Prior to analysis the data displaying a lack of normality were log transformed to stabilize variance. Data were analyzed using a two-way ANOVA with repeated measures for time. The sphericity adjustment was checked, and if required, a Greenhouse-Geisser epsilon correction was applied. Where no significant effect for treatment was observed, we explored pair-wise comparisons between individual time-points using the Least Significant Difference (LSD) of means. Bivariate relationships were examined with a Spearman rank correlation test. These statistical analyses were performed using GenStat for Windows 16th Edition (VSN International, Hemel Hempstead, UK). Confidence intervals were calculated at 90% using a bootstrapping technique for non-normally distributed data using SPSS Statistics Version 22 (IBM, Portsmouth Hampshire, UK). Statistical significance was set at *P* < 0.05.

## Results

### Muscle soreness

**Figure 1:**
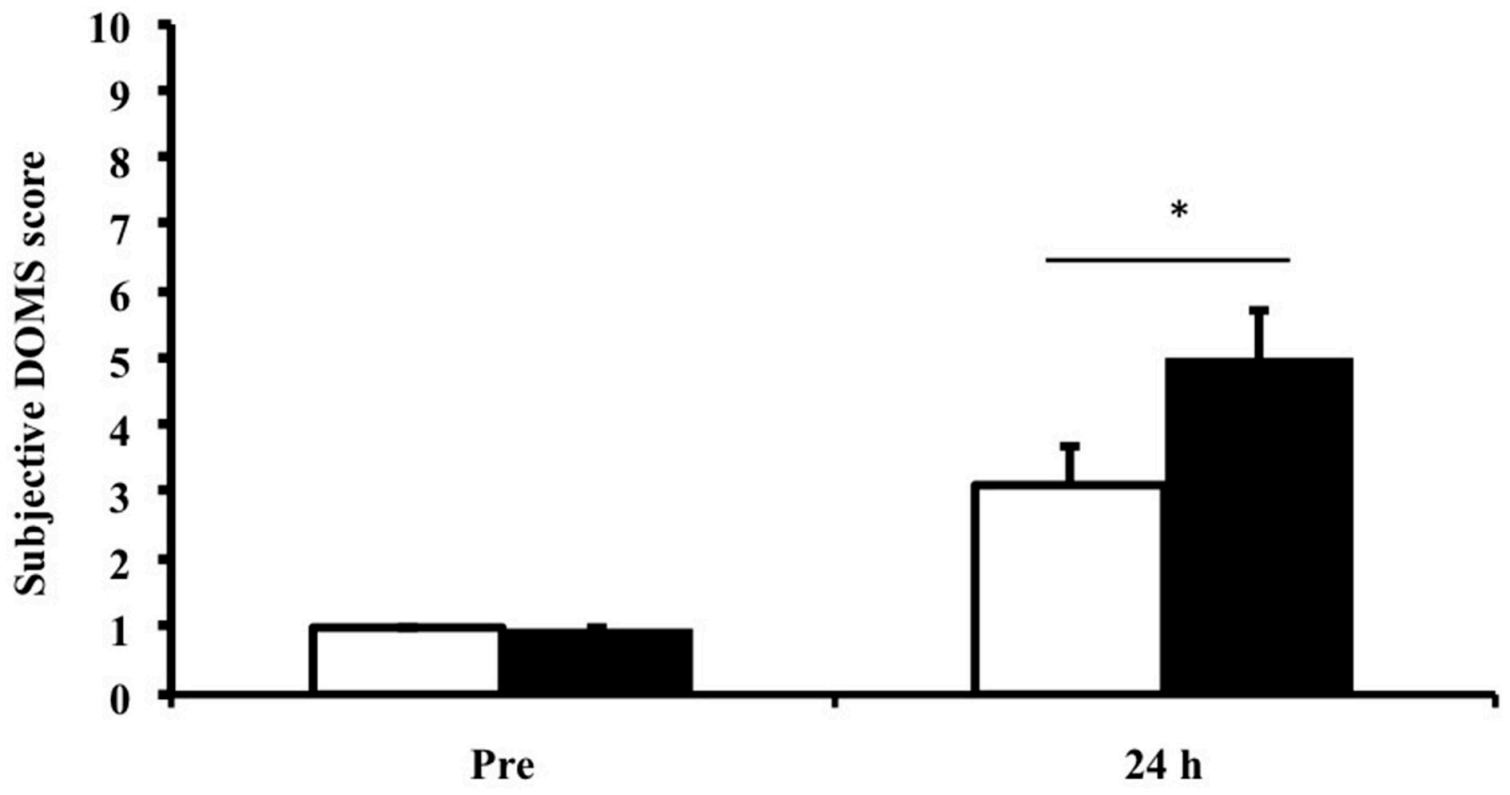
**Subjective rating for delayed onset muscle soreness (DOMS) Values depicted are mean values ± SEM**. ^*^denotes statistical significance from pre-exercise values (*p* < 0.05). White bars, PLA group; black bars, IBU group. This figure has been adapted from Vella et al. (2014).

Muscle soreness showed a main effect for time (*p* < 0.01), suggesting that the exercise protocol was sufficient to induce a muscle stress response. No effect of ibuprofen treatment was observed (Figure 1).

### Immunohistochemistry

**Figure 2:**
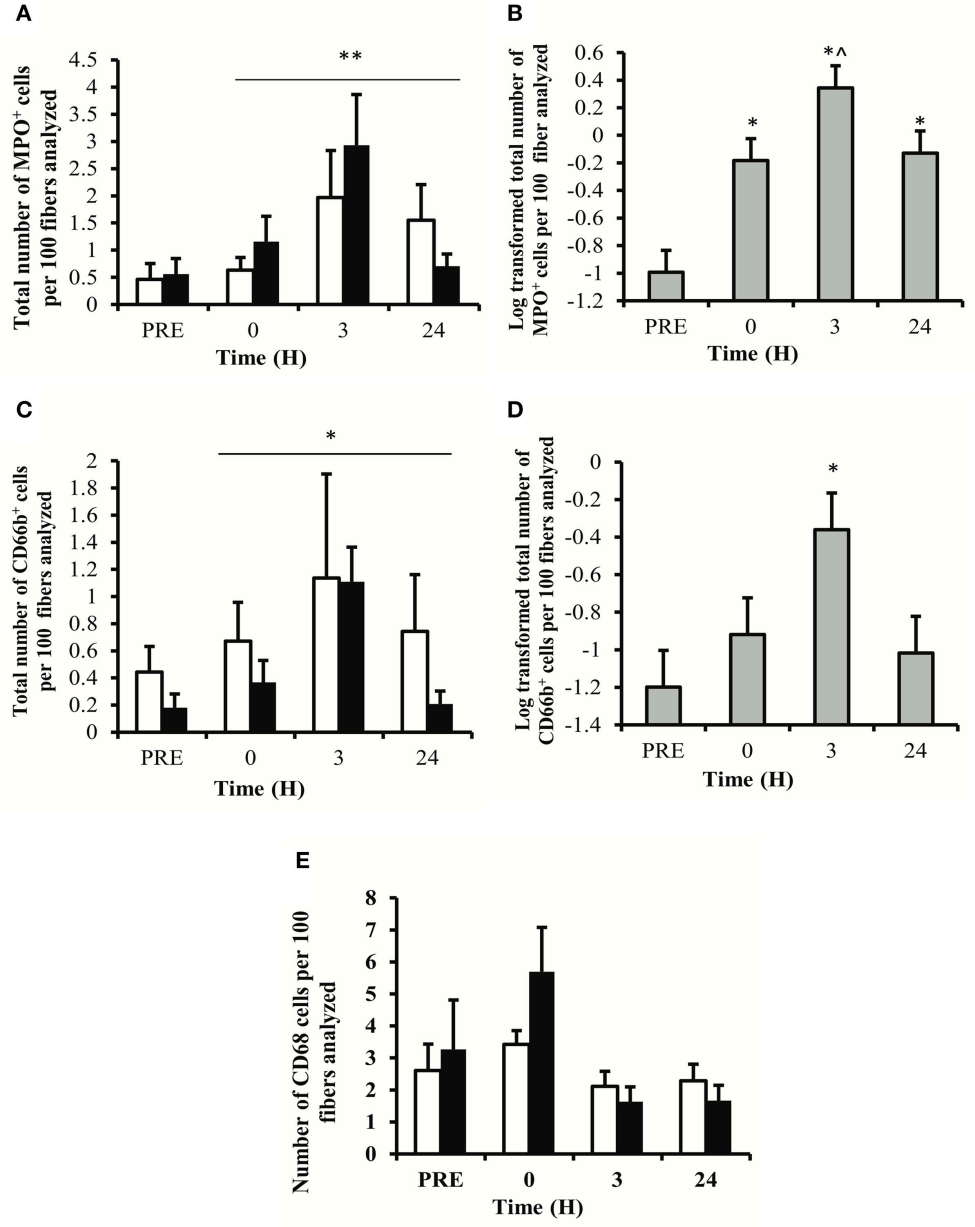
**Immunohistochemistry data for muscle cells staining positive for myeloperoxidase (MPO), showing group specific data (A) and collapsed data (B), CD66b showing group specific data (C), and collapsed data (D), and CD68 showing group data only (E)**. Data represent the mean number of positively stained cells per 100 fibers analyzed ± SEM. ^**^denotes statistical significance from pre-exercise values (*p* < 0.01). ^*^denotes statistical significance from pre-exercise values (*p* < 0.05). ^∧^denotes statistical significance from 0 h post-exercise (*p* < 0.05). White bars, PLA group; black bars, IBU group.

**Figure 3:**
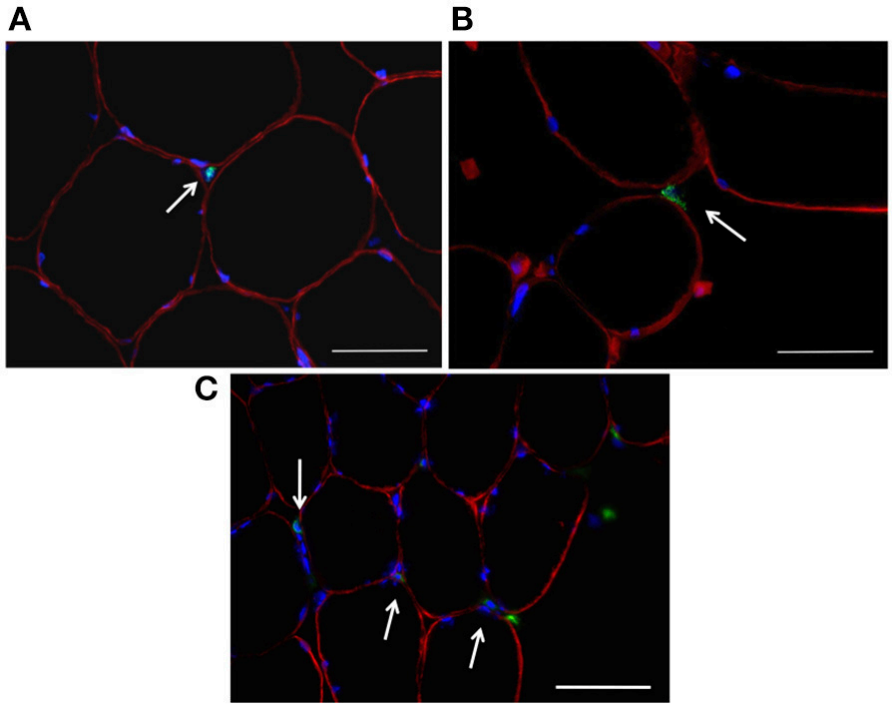
**Immunohistochemical analysis of skeletal muscle samples following about of resistance exercise**. Sections were probed with antibodies raised against leucocyte cell surface markers (green; **A**—MPO, **B**—CD66b, **C**—CD68) and dystrophin (red), while DAPI was used to stain nuclei (blue). Scale bars = 50 μm.

The number of MPO^+^ cells per 100 myofibers analyzed showed a main effect for time (*P* < 0.01). No main effect for treatment (*P* = 0.250) or time × treatment (*P* = 0.709) was observed (Figure 2A). LSD pairwise comparisons indicated a significant increase in MPO^+^ cells at all sampling time points post-exercise. The greatest increase in MPO^+^ cells was observed at 3 h post-exercise (Figure 2B). Similarly, the number of CD66b^+^ cells per 100 myofibers showed a main effect for time (*P* < 0.05), with no main effect for treatment (*P* = 0.149) or time × treatment interaction (*P* = 0.907; Figure 2C). LSD pairwise comparisons indicated a significant increase in the number of CD66b^+^ cells at 3 h post exercise (Figure 2D). The number of CD68^+^ cells per 100 myofibers analyzed showed no effect for time (*P* = 0.061), treatment (*P* = 0.530) or time × treatment interaction (*P* = 0.688; Figure 2E). MPO^+^, CD66b^+^, and CD68^+^ cells were observed within the endomysium and the perimysium, with no cellular infiltration occurring at any time point. Representative images for each cell surface marker are presented in Figure 3.

### Serum proteins

**Figure 4:**
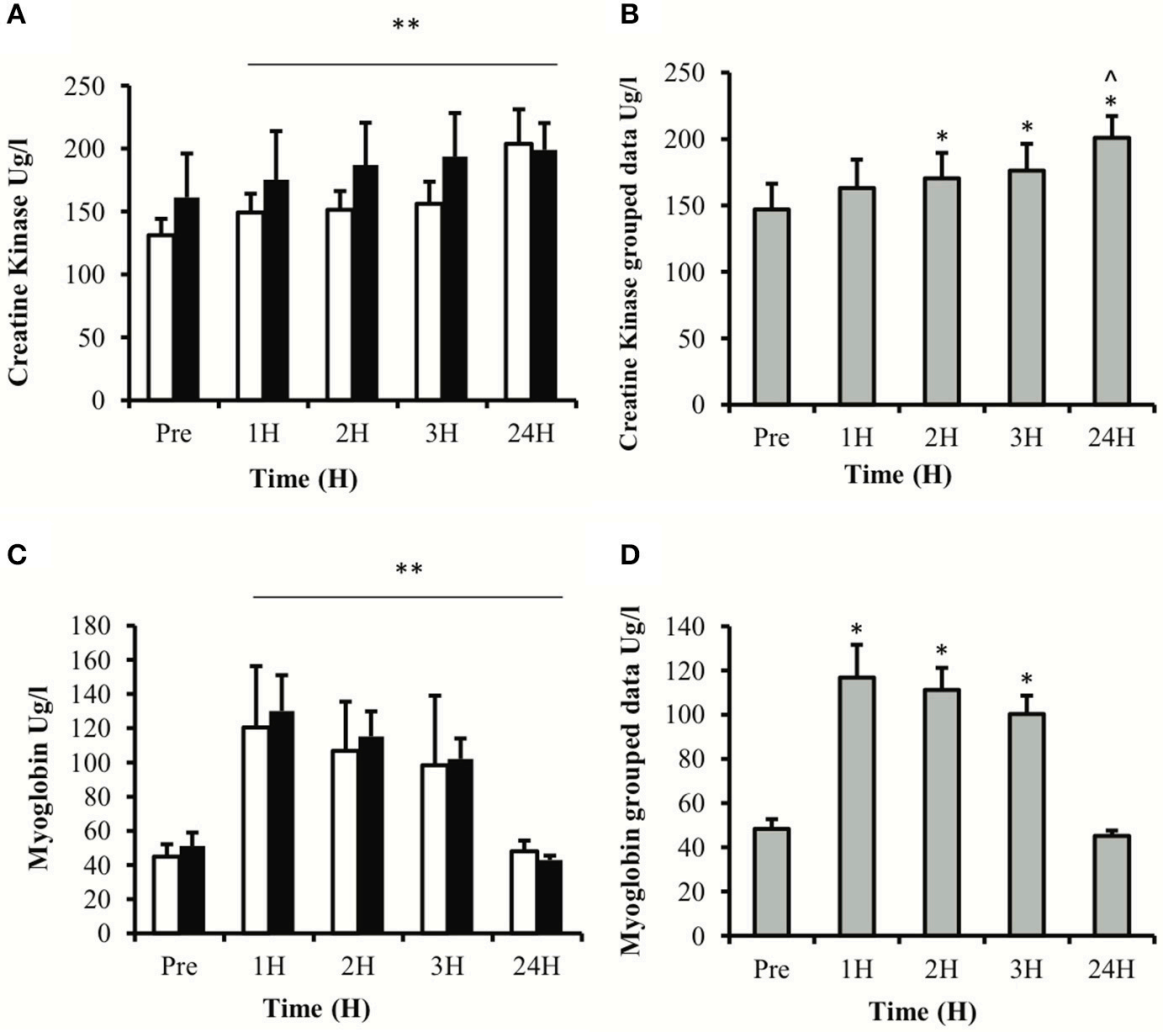
**Blood derived proteins creatine kinase (A,B) and myoglobin (C,D)**. Values depicted are mean values ± SEM. **(A,C)** represent data divided into two treatment groups; **(B**,**D)** represent collapsed data. ^*^denotes statistical significance from pre-exercise values (*p* < 0.05). ^**^denotes statistical significance from pre-exercise values (*p* < 0.01). ^∧^denotes statistical significance from 1, 2, and 3 h post exercise (*p* < 0.05). White bars, PLA; black bars, IBU.

Serum CK activity demonstrated a main effect for time (*P* < 0.01) with no effect for treatment (*P* = 0.843) or time × treatment interaction (*P* = 0.494; Figure 4A). LSD comparisons revealed that serum CK increased from pre-exercise values at 2 h post-exercise and peaked at 24 h post-exercise (Figure 4B). Similarly, serum myoglobin concentration showed a main effect for time (*P* < 0.01) with no effect for treatment (*P* = 0.710) or time × treatment interaction (*P* = 0.666; Figure 4C). LSD comparisons demonstrated that the biggest increase in serum myoglobin concentrations occurred at 1 h post-exercise and remained significantly elevated up to 3 h post exercise (Figure 4D).

### Correlation analysis

In general, large inter-individual differences were seen in the appearance of all of the leucocyte cell types. Results from the Spearman rank correlation test showed no correlation between the histochemical appearance of inflammatory leucocytes, biological markers of muscle damage, and subjective markers of muscle soreness (Table 2). Anecdotally, three subjects were identified as the highest responders in the appearance of cell surface markers applicable for localization of neutrophils (one subject within the placebo group, and two subjects within the ibuprofen group). However, these subjects showed no significant increase in CK, myoglobin or subjective markers of muscle damage when compared with the other subjects. Interestingly, subject 5 recorded the highest number of CD66b^+^ cells and MPO^+^ cells, whereas he presented with the lowest subjective rating for DOMS.

**Table 2:** **Spearman rank correlation analysis**.

	**CK**	**MYO**	**CD68^+^**	**CD66b^+^**	**MPO^+^**	**DOMS**
CK	–	0.59 (−0.01 − 0.91)	−0.46 (−0.88 − 0.8)	0.15 (−0.42 − 0.61)	0.36 (−0.14 − 0.73)	0.01 (−0.60 − 0.62)
MYO	–	–	0.01 (−0.55 − 0.52)	0.14 (−0.42 − 0.69)	0.35 (−0.21 − 0.67)	−0.33 (−0.74 − 0.24)
CD68^+^	–	–	–	0.05 (−0.46 − 0.56)	−0.27 (−0.75 − 0.33)	−0.37 (−0.82 − 0.25)
CD66b^+^	–	–	–	–	0.41 (−0.13 − 0.84)	−0.37 (−0.78 − 0.16)
MPO^+^	–	–	–	–	–	0.08 (−0.49 − 0.56)
DOMS	–	–	–	–	–	–

*Values depicted are the correlation coefficient with 90% confidence intervals in parenthesis. Statistical significance was set at p < 0.05. No statistically significant correlations were observed. CK, creatine kinase; MYO, myoglobin; MPO, Myeloperoxidase; DOMS, delayed-onset muscle soreness*.

## Discussion

The main finding of this study was that ibuprofen, a non-selective COX inhibitor, had no effect on the histological detection of leukocytes following an acute bout of traditional resistance exercise. Serum CK and myoglobin, and muscle soreness also did not change in response to ibuprofen. Furthermore, no correlation was observed between the accumulation of inflammatory leucocytes, increases in CK or myoglobin, and sensations of DOMS. These observations suggest that the intramuscular infiltration of inflammatory white blood cells, or an increase in serum levels of intramuscular proteins, are not predictive of post-exercise muscle soreness.

The current study focused on the acute local inflammatory response to exercise-induced muscle damage. Our protocol was effective in inducing muscle soreness as shown in Figure 1. It is important to note that we have previously reported this exercise-induced stress response (Vella et al., 2014). However, we felt it was necessary to re-emphasize this response here. In the present study we identified an increase in the histological appearance of CD66b^+^ and MPO^+^ cells that peaked at 3 h post exercise and found no effect of NSAID treatment. CD66b is a highly specific cell surface marker for detecting human neutrophils, and this is the first paper to show a small but significant increase in the intramuscular number of CD66b^+^ cells following acute resistance exercise. Paulsen et al. (2013) were unable to identify any change in the number of CD66b^+^ cells in the elbow flexors at 1, 2, 4, or 7 days following 70 maximal eccentric contractions (Paulsen et al., 2013). Differences in the timing of muscle biopsy samples may explain the discrepancy between the two trials, and suggest that neutrophils may be involved in the acute phase inflammatory response to resistance exercise. We also identified a small but significant increase in the number of MPO^+^ cells at all sampled time points, with a peak in expression at 3 h post exercise. MPO has typically been used to detect changes in neutrophils, and an increased number of MPO^+^ cells have been reported following high-force eccentric muscle contractions (Mahoney et al., 2008; MacNeil et al., 2011). However, MPO is also expressed on the lysosomes of monocytes and has been detected on basophils and eosinophils, perhaps providing a reason why there were a higher number of MPO^+^ cells when compared to CD66b^+^ cells detected in the present study (Paulsen et al., 2013).

No change was identified in the number of CD68^+^ cells. CD68 cell counts are widely used as a marker of monocyte/macrophage infiltration. However, CD68 may also be expressed on other cell types including satellite cells and fibroblasts (Paulsen et al., 2013). Other researchers have been able to identify a change in CD68^+^ cells following exercise-induced muscle damage (Peterson et al., 2003; Mahoney et al., 2008; MacNeil et al., 2011). Each of these studies have used a high-force maximal eccentric muscle contraction protocol, which may suggest that the detection of CD68^+^ cells is dependent on the extent of skeletal muscle damage.

Previous research exploring the effect on NSAIDs on post-exercise inflammation has produced equivocal findings. Earlier research from *in-vivo* rodent trials demonstrated a blunted inflammatory response to eccentric muscle contractions following the administration of both selective and non-selective NSAIDs (Lapointe et al., 2002a,b; Bondesen et al., 2004, 2006). However, these findings are yet to be supported in human trials (Bourgeois et al., 1999; Peterson et al., 2003; Tokmakidis et al., 2003). Peterson et al. (2003) found no effect of oral ibuprofen administration on the appearance of CD68^+^ cells 24 h following 100 eccentric muscle contractions (Peterson et al., 2003). Similarly, Tokmakidis showed no effect of ibuprofen on circulating white blood cells 24 h following an eccentric exercise protocol (Tokmakidis et al., 2003). Paulsen et al. (2010) proposed the concept of an inflammatory threshold, suggesting that NSAIDs may only influence post-exercise inflammation in response to a sufficiently strong inflammatory stimulus (Paulsen et al., 2010). It was suggested that the absence of an intramuscular prostaglandin response to exercise, specifically PGE_2_, could explain the lack of an NSAID effect on leucocyte recruitment (Trappe et al., 2001; Paulsen et al., 2010). Interestingly, our group recently reported an increase in serum PGE_2_ in samples that were obtained during the same exercise trials as those currently reported (Markworth et al., 2013). This change in serum PGE_2_ was blunted by the administration of ibuprofen and suggests that in the presence of a blunted prostaglandin response to exercise, NSAID treatment had no effect on leucocyte recruitment within skeletal muscle. Although this finding does not refute the possibility of an inflammatory threshold, based on our previous findings (Markworth et al., 2013), it is reasonable to suggest that the presence of an exercise-induced serum prostaglandin response is not the required underlying mechanism for neutrophil infiltration of muscle during the early hours of post-exercise recovery.

In accordance with previous studies, resistance exercise resulted in an increase in serum CK and myoglobin, and subjective muscle soreness (Nosaka et al., 2006). Although large inter-individual variations in the human response to exercise can make it difficult to establish causal relationships, there was no relationship between the appearance of leucocytes and blood-derived markers of muscle damage or sensations of muscle soreness. Interestingly, the subject with the highest number of CD66b^+^ and MPO^+^ cells presented with the lowest subjective rating of DOMS. These findings provide support for the assumption that leucocyte recruitment and inflammation do not consistently cause DOMS.

From animal studies it appears that cellular events in post-exercise inflammation, occur concomitantly with the onset of DOMS (Armstrong et al., 1991; Faulkner et al., 1993). The mechanistic link has been proposed to be a leucocyte-mediated release of cytotoxic substances and reactive oxygen species as a bi-product of phagocytosis (Connolly et al., 2003). Furthermore, the prostaglandin response to exercise has been shown to occur concurrently to the onset of DOMS, and is known to influence both the recruitment of inflammatory leucocytes and neural afferents to pain (Markworth et al., 2013). However, in line with the current findings, the accumulation of inflammatory leucocytes in human skeletal muscle tissue during exercise recovery appears to be both spatially and temporally out of phase with the development of DOMS (Paulsen et al., 2010). Malm et al. (2004) explored the concept that the activation of local leucocytes present in the epimysium may be involved in the regulation of DOMS. This hypothesis has since received support from both rodent (Gibson et al., 2009) and human trials (Crameri et al., 2007; Raastad et al., 2010) and presents a promising area for future research.

## Conclusion

This is the first study to demonstrate that ibuprofen administration has no effect on the histological appearance of inflammatory white blood cells following an acute bout of traditional resistance exercise. We also found no effect of ibuprofen administration on blood markers of muscle damage or subjective muscle soreness, and no significant correlations between leucocyte numbers and post-exercise muscle damage or soreness. Future research needs to explore the possibility of an inflammatory threshold above which NSAID administration influences post-exercise inflammation. The underlying cause of post-exercise muscle soreness also remains largely unknown. Future investigations are needed to determine the role that inflammation plays in exercise-induced DOMS.

## Author contributions

LV—Research design, sample acquisition and analysis, data analysis, drafting and formatting manuscript. JM—Research design, data analysis, revising manuscript, final approval of version to be published. GP—Data collection and analysis, revising manuscript, final approval of version to be published. TR—Data collection and analysis, interpretation of data, revising manuscript, final approval of version to be published. JP—Research design, data analysis, interpretation of data, revising manuscript, final approval of version to be published. RS—Research design, interpretation of data, revising manuscript, final approval of version to be published. DC—Research design, interpretation of data, revising manuscript, final approval of version to be published. AR—Data analysis, interpretation of data, revising manuscript, final approval of version to be published.

### Conflict of interest statement

The authors declare that the research was conducted in the absence of any commercial or financial relationships that could be construed as a potential conflict of interest.
